# Physiological complexities of intraabdominal hypertension: taming the elephant

**DOI:** 10.1186/s13613-025-01481-9

**Published:** 2025-05-31

**Authors:** S Magder

**Affiliations:** Critical Care and Cardiology, McGill University Health Centre, 1001 Decrie Blvd, Montreal, QC H4A 3J Canada

I thank Dr Triu [1] for his kind words about my paper on Guyton and fluid management [2]. Intra abdominal hypertension (IAP) is an interesting pathophysiological problem. It was more than I wanted to discuss in my introductory paper. However, I cannot resist the challenge to indulge in some physiological speculations, my favourite past-time! I basically agree with Dr Triu’s interpretation [2], but I would like to add some additional thoughts that I hope will be helpful.

His statement that the system should not be broken into components should be considered a fundamental rule when evaluating the circulation. The system is a closed loop circuit and conservation of mass must always be considered. Some general rules. In the steady state, stressed volume is constant. Resistances between compartments determine where volume distributes, but the pressure in each region is determined by the volume it contains, its local compliance, and how it empties. Thus, a volume shift from the very compliant systemic venous compartment to the less compliant pulmonary veins produces a seven-fold higher rise in pulmonary venous pressure than the fall in systemic venous pressure and contributes to pulmonary edema with IAP.

Personally, I think that the primary pathophysiology of abdominal compartment syndrome is not complicated. As noted by Dr Triu, the primary mechanism is the creation of “waterfall” or zone II conditions in the abdomen as elegantly studied by Takata et al [3]. The collapse pressure means that upstream splanchnic venous pressure must be higher to maintain the necessary pressure difference for venous return. Actual venous resistance likely, too, is increased by the increased IAB. Thus, an adequate driving pressure for venous return requires giving more volume to raise MSFP. However, this increases the upstream systemic capillary pressures and increases capillary filtration and intra-abdominal volume. This further increases intra-abdominal pressure, further compresses abdominal veins, and the vicious cycle goes on. As a simple point, volume in abdomen cannot increase if the clinician does not give the volume! Abdominal volume does not come out the air! Treating the elephant in the room is simple. Stop giving volume, get a surgeon to open and decompress the abdomen, resect ischemic bowel and drain accumulating collections questions.

I think that is dominant process. However, interactions between the abdomen and chest bring up some other interesting physiology. In the preface to his book The Thorax, my mentor Dr D Roussos, noted that in Greek, the thorax goes from the sternal notch to the symphysis pubis [4]. Thus, the chest and abdomen are connected, and increased abdominal pressure is transmitted to the chest, especially as the abdominal wall gets progressively less compliant and more like the chest wall.

The rise in right atrial pressure (RAP) with IAP is not paradoxical if one separates transmural RAP from RAP relative to atmosphere. Transmural RAP and volume decrease because of the upstream flow limitation and lower cardiac output. However, increased RAP relative to atmosphere is important, too, because it is the back pressure for venous return. A rise in this pressure further increases systemic capillary leak [5].

I do not consider a decrease in left ventricular compliance a major issue. Left ventricular SV must be the same as RVSV. The left atrial pressures, though, may be higher and produce more pulmonary capillary leak.

The effect of all this on the RV is very complicated and can become important if RV functions is depressed. I confess that what I am writing here is very speculative because of all the interacting factors. First, one must consider whether ventilation is spontaneous (“negative pressure” inspiration) or positive pressure inspiration [6]. The IAB decrease in thoracic wall compliance requires larger inspiratory muscle efforts, which could lead to non-West conditions in the pulmonary circuit and increase the load on the RV. Because of the decreased chest compliance from the transmitted abdominal pressure, the inspiratory rise in pleural pressure with a mechanical breath is greater and this can further increase upstream venous pressures and worsen capillary, leak as well as the RV load from a rise in pulmonary pressure.

In summary, the dominant process in IAP is the requirement for higher systemic venous pressure to maintain adequate RV diastolic filling. Other factors create complicated interacting factors that are fun for physiological speculation, but management still is simple. Stop giving more fluids and decompress the abdomen!!

## Data Availability

NA
